# A 40-Year-Old Male Presenting with Hereditary Multiple Exostosis: Management and Considerations

**DOI:** 10.1155/2019/4793043

**Published:** 2019-03-13

**Authors:** Matthew Wells, Zackary Birchard

**Affiliations:** ^1^Lake Erie College of Osteopathic Medicine, OMSIII, USA; ^2^Lake Erie College of Osteopathic Medicine, D.O., M.S.B.S, 5515 Peach Street, Erie, PA 16509, USA; ^3^Department of Orthopedic Surgery, Millcreek Community Hospital, 5515 Peach Street, Erie, PA 16509, USA

## Abstract

Hereditary multiple exostosis is a rare condition in which numerous benign osteochondromas form throughout the body, typically in areas of high bone turnover such as the metaphyseal plates of long bones. While many of these lesions remain clinically asymptomatic, other growth locations can lead to excruciating pain, limit a joint's range of motion, and compromise neurovascular structures. These patients undergo multiple surgeries throughout their lifetime in order to remove symptomatic osteochondromas. Due to deformities and changes in bone structure, these patients also suffer from significant arthritis which may also require surgery. It is important that a skilled orthopedic surgeon follow these patients in order to help them make informed decisions and limit the number of surgeries within their lifetime. The purpose of this case report is to discuss one instance in which a patient's significant arthritis was operatively managed in the setting of hereditary multiple exostosis.

## 1. Introduction

Hereditary multiple exostosis (HME) is a rare inherited genetic condition characterized by the presence of multiple benign osteochondromas (exostoses) that affect roughly 1 in 50,000 people and does not appear to have a sexual predominance [1, 2]. HME displays an autosomal dominant inheritance pattern and is predominantly caused by loss of function of two genes: exostosin-1 (EXT1) located on chromosome 8 or exostosin-2 (EXT2) located on chromosome 11 [3]. HME is genetically heterogeneous as both EXT1 and EXT2 serve as tumor-suppressor genes of the EXT gene family. All members of this multigene family encode for glycosyltransferases involved in the adhesion and polymerization of heparan sulfate (HS) chains as they are transported through the endoplasmic reticulum and Golgi system [4]. Heparan sulfate is an essential component of a cell's surface and extracellular matrix. Studies have indicated that the heparan sulfate chains help form the proteoglycan matrix that serves to regulate the distribution and availability of growth and signaling proteins and their respective interactions, function, and bioactivity on target cells. Many of these signaling proteins include the members of the hedgehog, bone morphogenetic protein (BMP), fibroblast growth factor (FGF), and Wnt families. Consequently, HS has many important processes in skeletal tissue genesis and growth, especially within the metaphyseal growth plate [5]. The heterogeneity of EXT1 and EXT2 follow Knudson's two-hit model for tumor suppressor genes as multiple osteochondromas arise via a second-hit mutation. This has been shown in 63% of analyzed osteochondromas in which the detection of the second hit was demonstrated through the ratio of HS-positive (normal) versus HS-negative (mutated) cells in the cartilaginous cap of osteochondromas. These findings were displayed in both the sporadic single osteochondromas and the multiple found in HME [6]. However, the exact biochemical mechanism for multiple osteochondroma formation is yet to be elucidated.

Due to their location, size, number, and interactions, the osteochondromas can cause compression of nerves, blood vessels, and tendons with consequential pain and impairment of motion. They also lead to skeletal deformities and growth restriction by interfering with normal growth plate function. Most patients with HME often show slightly shortened stature, bowing and shortening of the forearms, changes in angulations of the knee and fingers, and limb-length inequalities [7]. Currently, surgery is the most common treatment for HME patients by which the most symptomatic, problematic, and accessible osteochondromas are resected in order to ameliorate major skeletal defects. However, this can have a significant effect on the patients' mental and physical well-being. To put it into perspective, D'Ambrosi et al. followed 50 patients with HME who had a mean number of 18.12 osteochondromas per individual, and each patient had undergone a mean of 5.62 surgical procedures for osteochondroma removal [8].

Unfortunately, osteochondromas may be difficult to reach or are located in potentially dangerous and delicate locations; as a consequence, many are often left in place, leading to long lasting problems and concerns [9]. While early onset osteoarthritis is common, the most feared complication of HME is transformation of a benign osteochondroma into a malignant chondrosarcoma at a rate of 2-5% of patients [10]. Regardless of their underlying hereditary diagnosis, these patients still experience the same joint degeneration as any other aging individual. This case presentation seeks to discuss the intricate role of orthopedic surgeons in limiting the number of procedures patients with HME undergo while still managing the chronic arthritis that is prevalent within the population.

## 2. Case Presentation

**Figure 1:**
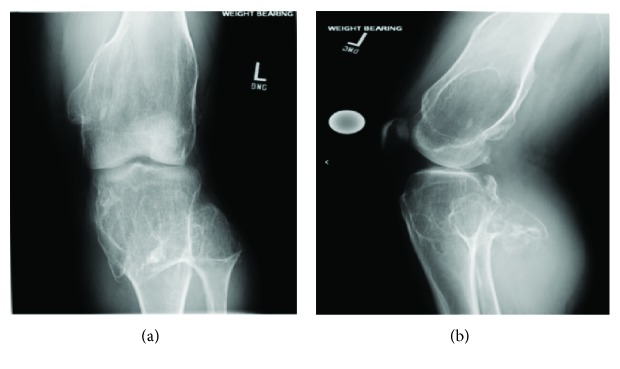
A preoperative anterior-posterior (AP) and lateral X-ray view of the patient's left knee showing significant osteochondroma formation both superior and inferior to the knee joint. Subchondral sclerosis as well as medial and lateral loss of joint space is appreciable.

A 40-year-old male presented to our office in 2017 complaining of chronic left knee pain. This was his first visit to this office and was establishing care after moving to the area. The patient's electronic medical record was obtained from an outside institution which indicated a past medical history of diabetes, hyperlipidemia, and hereditary multiple exostosis. The patient disclosed that he had multiple osteochondroma removal surgeries which included his left knee, lumbar spine, and left foot at an outside institution. In addition, he had a left hip arthroplasty with refractory numbness/tingling of the leg. The patient stated he had left knee pain for years until he had an osteochondroma removed in his left distal femur in 2016 which seemed to help for 6 months. He stated the pain returned at 7/10 and is worse with movements. He had limited range of motion with 70° of flexion, negative pain with varus and valgus movement, and negative secondary tests. Baseline X-rays were ordered for this patient's knee (Figure 1).

**Figure 2:**
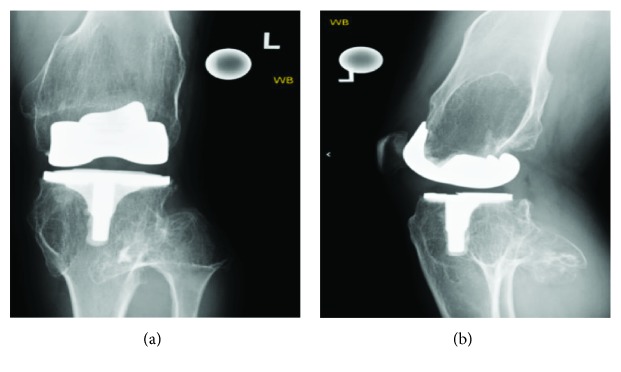
A two-month postoperative AP and lateral X-ray view of the patient's left knee displaying a Smith & Nephew posterior stabilized prosthesis with unchanged osteochondroma formation.

There were osteoarthritic changes with significant osteochondroma formation; however, arthroplasty was not recommended at that time because of the patient's young age and his left lower extremity being distally neurovascular intact. The patient was adamant about having his knee replaced and was referred to a specialist at an outside institution where an arthroplasty of the left knee was performed. The patient returned 3 months later with continued limited active and passive range of motion (<90° flexion). Follow-up radiographs were ordered showing good alignment of a Smith & Nephew posterior stabilized prosthesis without subsidence (Figure 2). It was decided to perform manipulation under anesthesia followed by 6 weeks of physical therapy which improved his range of motion to 110° flexion.

**Figure 3:**
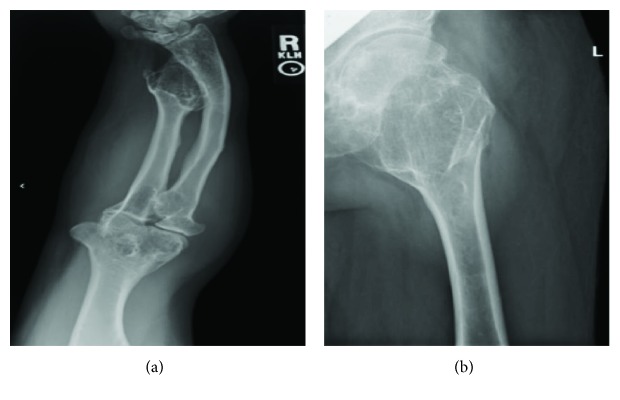
(a) An AP view of the patient's right forearm with multiple osteochondroma formations, cubitus varus deformity, and shortened limb length. (b) An AP view of the patient's left femur displaying a large femoral neck osteochondroma with deformity of trochanteric structures.

**Figure 4:**
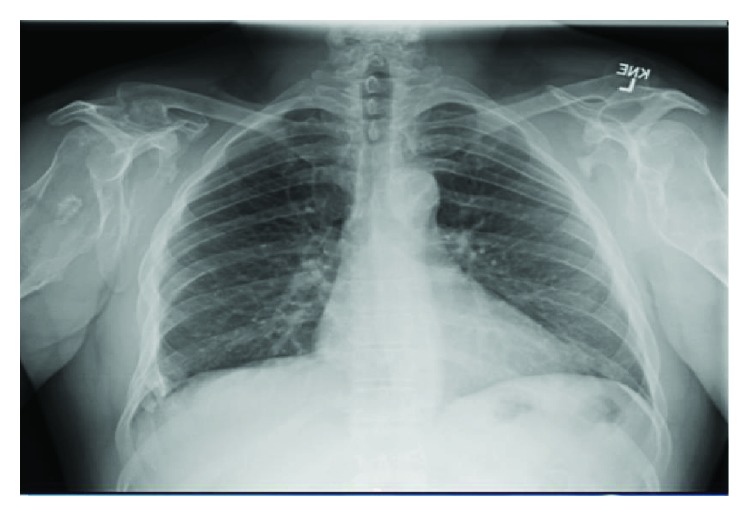
An AP X-ray view displaying multiple osteochondromas at multiple sites in the clavicles, scapulae, and proximal humeri bilaterally as well as multiple right inferior anterior ribs.

The patient continues to have limited range of motion of his left knee with flexion to 90°. The large posteriorly projecting osteochondroma of his left tibia continues to be asymptomatic and painless, and therefore, it was decided not to perform surgical interventions at this time. Performing an arthroplasty helped relieve the patient's pain, however, did not result in significant improvement of active and passive range of motion of the joint. Overall, the patient is satisfied with his knee replacement but still has refractory numbness/tingling of his distal left lower extremity from his prior hip replacement. The patient has chronic pain elsewhere, which is being closely monitored (Figures 3 and 4).

## 3. Discussion

HME is a difficult disease to manage and has variable presentation between each patient. It is suggested to refrain from surgery until individuals with HME achieve skeletal maturity for fear of recurrence in the skeletally immature patient. Once the patient reaches skeletal maturity, the physician should feel more comfortable in performing osteochondroma removal. Given the deformities seen in these patients, arthritis can develop at younger ages presenting as lower reported sports activities in children and the need for earlier intervention than the average population [11]. In this instance, our patient received a left total knee arthroplasty which relieved his arthritic pain, however, did not significantly improve his active or passive range of motion. As seen on radiographs, this patient currently has a significantly large posteriorly projecting osteochondroma on his left proximal tibia that would alarm most physicians given the significant neurovascular structures within the same anatomic area. However, given this patient's extensive past surgical history and continued management, it was decided not to remove this osteochondroma until it becomes symptomatic. This case highlighted the importance of close monitoring of patients with HME and how changes in bone structure can lead to early onset arthritis requiring intervention. This case served to reinforce the conservative approach of removing osteochondromas only if medically necessary while maximizing the quality of life and minimizing the pain of associated arthritis seen in patients suffering from HME.

## 4. Conclusion

Patients who suffer from hereditary multiple exostosis will require multiple surgeries throughout their lifetime in order to intervene against symptomatic exostoses and debilitating arthritis. Given the increased risk of adverse side effects secondary to surgical procedures, it is necessary to construct an agreeable plan to refrain from osteochondroma removal unless medically necessary.
